# Frequent somatic *TERT* promoter mutations and *CTNNB1* mutations in hepatocellular carcinoma

**DOI:** 10.18632/oncotarget.12121

**Published:** 2016-09-19

**Authors:** Seung Eun Lee, Seong-Hwan Chang, Wook Youn Kim, So Dug Lim, Wan Seop Kim, Tea Sook Hwang, Hye Seung Han

**Affiliations:** ^1^ Department of Pathology, Konkuk University Medical Center, Konkuk University School of Medicine, Seoul, Korea; ^2^ Department of Surgery, Konkuk University School of Medicine, Seoul, Korea

**Keywords:** TERT, CTNNB1, hepatocellular carcinoma, intratumoral genetic heterogeneity, Pathology Section

## Abstract

Genetic alterations of *TERT* and *CTNNB1* have been documented in hepatocellular carcinoma. *TERT* promoter mutations are the earliest genetic events in the multistep process of hepatocarcinogenesis related to cirrhosis. However, analyses of *TERT* promoter and *CTNNB1* mutations in hepatocellular carcinoma tumor samples have not been performed in the Korean population, where hepatitis B virus-related hepatocellular carcinoma is prevalent. In order to identify the role of *TERT* promoter and *CTNNB1* mutations in the hepatocarcinogenesis and pathogenesis of recurrent hepatocellular carcinoma, we performed the sequence analyses in 140 hepatocellular nodules (including 107 hepatocellular carcinomas), and 8 pairs of matched primary and relapsed hepatocellular carcinomas. *TERT* promoter and *CTNNB1* mutations were only observed in hepatocellular carcinomas but not in precursor lesions. Of 109 patients with hepatocellular carcinoma, 41 (39.0%) and 15 (14.6%) harbored *TERT* and *CTNNB1* mutations, respectively. *TERT* promotermutations were significantly more frequent in hepatocellular carcinomas related to hepatitis C virus infection (5/6; 83.3%) compared to tumors of other etiologies (*P* = 0.001). In two cases, discordance in *TERT* promoter mutation status was observed between the primary and the corresponding recurrent hepatocellular carcinoma. The two patients with discordant cases had early relapses. In conclusion, we identified *TERT* promoter and *CTNNB1* mutations as the most frequent somatic genetic alterations observed in hepatocellular carcinoma, indicating its pivotal role in hepatocarcinogenesis. Furthermore, we suggest the possibility of intratumoral genetic heterogeneity of *TERT* promoter mutations in hepatocellular carcinoma as indicated by the discordance in *TERT* promoter mutations between primary and corresponding recurrent hepatocellular carcinoma.

## INTRODUCTION

Hepatocellular carcinoma (HCC) is the fifth most common cancer and the third leading cause of cancer-related death worldwide. Despite recent advances in therapeutic strategies for the treatment of HCC, it still has a poor prognosis. The recurrence of HCC after resection remains the major cause of death [1, 2]. Patients with HCC have high recurrence rate and intrahepatic recurrence occurs in 70-100% of cases within 5 years after hepatic resection for HCC [3–5].

The majority of HCCs develop in liver cirrhosis related to chronic hepatitis B virus (HBV) infection, chronic hepatitis C virus (HCV) infection, alcohol intake, and obesity. HCC is considered to develop in a multistep process, in which a precursor or premalignant lesion progresses to dysplastic nodules, followed by HCC. Hepatocarcinogenesis has been characterized as the progressive accumulation of a variety of genetic alterations in chronic liver disease. It is well known that HCCs are phenotypically and genetically heterogeneous tumors. A detailed understanding of the genomic alterations in HCC can improve tumor characterization to help identify molecular targets for therapies. In particular, knowledge of the molecular mechanisms of tumor progression could be of potential value for therapeutic decisions.

Recently, genetic alterations of *TERT* have been documented in HCC [6, 7]. *TERT* gene alterations identified by exome sequencing in the International Cancer Genome Consortium (ICGC) and The Cancer Genome Atlas (TCGA) occur in more than 68% of HCCs and were ancestry-independent [6]. Of note is that somatic mutations in the *TERT* promoter have been frequently identified in HCC [8]. The frequency of *TERT* promoter mutations in HCC has been reported to be 44~59% [8–10]. In particular, it has been reported that *TERT* promoter mutations are the earliest genetic events in the multistep process of hepatocarcinogenesis related to cirrhosis [8]. *TERT* promoter mutations include 2 hot spots (C228T and C250T) that were identified in various types of tumors. *TERT* promoter mutations were first reported in melanoma [11], and were subsequently identified in urothelial carcinoma, glioma, and papillary thyroid carcinoma [12–14]. *TERT* is the catalytic subunit of the telomerase complex, and is a predominant determinant for controlling telomerase activity. Telomerase plays a key role in increasing the longevity of cells by maintaining the length of telomere caps at the end of chromosomes. Telomerase activation is involved in mechanisms of tumorigenesis and telomerase activity is actually upregulated in 85~90% of cancers [15]. The mechanisms of telomerase reactivation in cancer have yet to be fully explored. Activating mutations in the promoter of the *TERT* gene leads to increased telomerase expression.

A critical role of *CTNNB1* (a gene encoding β-catenin) mutations in hepatocarcinogenesis has been established [16–18]. *CTNNB1* mutations are among the most frequent genetic alterations in HCC and have been reported in 20~40% of cases. *CTNNB1* mutations are more common in HCV-related HCCs compared to HBV-related HCCs [17]. Furthermore, hepatocellular adenomas (HCAs) harboring *CTNNB1* mutations are more at risk of malignant transformation leading to the development of HCC [19]. These mutations predominantly occur within exon 3 of the gene, in a region encoding for the protein sequence containing the consensus sites for phosphorylation, and prevent β-catenin from phosphorylation and subsequent degradation. Recently, it has been reported that *TERT* promoter mutations and *CTNNB1* mutations in hepatocellular tumors are significantly associated [8].

The analyses of *TERT* promoter and *CTNNB1* mutations on HCC tumor samples have not been performed in the Korean population, where HBV-related HCC is prevalent. In order to identify the role of *TERT* promoter mutations and *CTNNB1* mutations in hepatocarcinogenesis and the pathogenesis of recurrent HCC, we performed the mutational analyses in full spectrum of precancerous lesions and HCC and in 8 pairs of matched primary and relapsed HCCs.

## RESULTS

### Prevalence of TERT C228T/C250T and CTNNB1 mutations in hepatocellular nodules

A total of 156 liver nodules were evaluated, including 4 LRNs, 10 LDNs, 1 HDNs, 9 HCAs, 123 HCCs, and 3 combined HCCs and cholangiocarcinomas. Sequence analyses of the *TERT* promoter and *CTNNB1* exon 3 were performed for 123 HCCs, of which 119 and 116 had successful results, respectively. The mutation frequency in each hepatocellular nodule is shown in Table 1. *TERT* promoter mutations were only observed in HCCs. *TERT* promoter and *CTNNB1* mutations were present in 37.8% (45 of 119) and 13.8% (16 of 116) of HCCs, respectively. All *TERT* promoter mutations were found at 2 hotspots (C228T and C250T). Among 45 *TERT* mutant cases, only 2 had C250T, while the rest had C228T. *TERT* promoter mutations at the 2 hotspots were mutually exclusive.

In *CTNNB1* mutation analysis, 16 out of 116 HCCs (13.8%) had missense point mutations. These mutations were C86T (S29F) in 2 cases, C98G (S33C) in 2 cases, A95C (D32A) in 1 case, G94A (D32N) in 1 case, A95T (D32V) in 1 case, A95G (D32G) in 1 case, C134T (S45F) in 3 cases, T133G (S45A) in 1 case, T109G (S37A) in 1 case, T133C (S45P) in 2 cases, and A121G (T41A) in 1 case. No *TERT* and *CTNNB1* mutations were identified in preneoplastic lesions including 10 LDNs and 7 HDNs, as well as 4 LRNs.

We additionally examined 9 HCAs without malignant transformation including 3 β-catenin-activated HCAs showing nuclear staining of β-catenin. Only 1 case harbored *CTNNB1* mutations; however, no *TERT* promoter mutations were observed in 9 HCAs. There were no mutations in 3 combined HCCs and cholangiocarcinomas.

**Table 1:** Frequency of *TERT* promoter and *CTNNB1* mutations in hepatocellular nodules

		TERT C228T & C250T status		CTNNB1 status	
	Total	Wild type	Mutant	Wild type	Mutant
	(*n* = 156)	(*n* = 107)	(*n* = 45)	(*n* = 127)	(*n* = 17)
**Hepatic adenoma**	9	9 (100%)	0	8 (88.9%)	1 (11.1%)
**Regenerating nodule**	4	4 (100%)	0	2 (100%)	0
**Low grade dysplastic nodule (Large cell change)**	10	10 (100%)	0	8 (100%)	0
**High grade dysplastic nodule**	7	7(100%)	0	6 (100%)	0
**Hepatocellular carcinoma**	123	74 (60.2%)	45 (36.6%)	100 (86.2%)	16 (13.8%)
**Combined hepatocellular carcinoma and cholangiocarcinoma**	3	3 (100%)	0	3 (100%)	0

### Clinicopathological demographics of the patients with HCC

A total of 123 HCC samples from 109 patients were analyzed in this study.

The study population consisted of 95 men (87.2%) and 14 women (12.8%), with a median age of 55 years (range, 27-81). Seventy-seven patients (70.6%) were younger than 60 years, and 32 patients (29.4%) were older than 60 years of age. Of these, 61 patients (56.0%) had underlying liver cirrhosis. All patients had chronic liver disease related to HBV infection (80/109, 73.4%), HCV infection (7/109, 6.4%), alcohol consumption (9/1109, 8.3%), and unknown causes (13/109, 11.9%). Of these, 101 patients (92.7%) underwent liver resection and 8 patients (7.3%) underwent liver transplantation. Median follow-up duration was 23 months (range, 0-115 months) after the operation. Of 109 patients, recurrence and death occurred in 37 patients (33.9%) and 12 patients (11.0%), respectively.

### Association between TERT / CTNNB1 mutations and clinicopathological features of HCC

Of 109 patients with HCC, 41 (39.0%) and 15 (14.6%) harbored *TERT* and *CTNNB1* mutations, respectively. The association between *TERT*C228T/*CTNNB1* mutations, and clinicopathological factors in HCC patients was evaluated (Table 2).

*TERT* promoter mutations were significantly more common in HCCs related to HCV infection (5/6; 83.3%) compared to tumors of other etiologies (*P* = 0.001). The frequency of *TERT* promoter mutations was 29.5% (23/78) in HCC related to HBV infection. However, there was no significant difference in *TERT* mutation status regarding sex, age at diagnosis, presence of underlying liver cirrhosis (LC), tumor size, tumor multiplicity, presence of vascular invasion, ES grade, T stage, and AJCC stage. Rates of recurrence and death did not differ based on *TERT* mutation status.

*CTNNB1* mutations were not associated with any clinicopathological factors. *CTNNB1* mutations were present in 13.2% (10/76) of HCC related to HBV, and in 14.3% (1/7) HCC related to HCV. Rates of recurrence and death did not differ based on *CTNNB1* mutation status.

*TERT* promoter mutations were found in 46.7% (7/15) and 38.8% (33/85) of *CTNNB1* mutant and wild-type *CTNNB1* cases, respectively. Conversely, *CTNNB1* mutations were found in 17.5% (7/40) and 13.3% (8/60) of *TERT* promoter mutant and wild-type *TERT* tumors, respectively. There was no significant association between *TERT* promoter mutations and *CTNNB1* mutations (*P* = 0.568).

**Table 2:** Clinicopathologic factors according to *TERT* promoter and *CTNNB1* mutation status

		Total (*n*=105)	*TERT* mutated (*n*=41, 39.0%)	Wild *TERT* (*n*=64, 61.0%)	*P*-value	Total (*n*=103)	*CTNNB1* mutated (*n*=15, 14.6%)	Wild *CTNNB1* (*n*=88, 85.4%)	*P*-value
Age			60 ±10.4	54±10.3	0.007		59 ±10.6	56±10.6	0.225
Sex	Male	92 (87.6%)	38 (92.7%)	54 (84.4%)	0.207	90 (87.4%)	14 (93.3%)	76 (86.4%)	0.686
	Female	13 (12.4%)	3 (7.3%)	10 (15.6%)		13 (12.6%)	1 (6.7%)	12 (13.6%)	
Aetiology	HBV	78 (74.3%)	23 (56.1%)	55 (85.9%)	**0.001**	76 (73.8%)	10 (66.7%)	66 (75.0%)	0.747
	HCV	6 (5.7%)	5 (12.2%)	1 (1.6%)		7 (6.8%)	1 (6.7%)	6 (6.8%)	
	Alcohol	8 (7.6%)	3 (7.3%)	5 (7.8%)		7 (6.8%)	1 (6.7%)	6 (6.8%)	
	Unknown	13 (12.4%)	10 (24.4%)	3 (4.7%)		13 (6.8%)	3 (20.0%)	10 (11.4%)	
LC	Yes	58 (55.2%)	24 (58.5%)	34 (53.1%)	0.688	60 (58.3%)	10 (66.7%)	50 (56.8%)	0.475
	No	47 (44.8%)	17 (41.5%)	30 (46.9%)		43 (41.7%)	5 (33.3%)	38 (43.2%)	
Tumor size	<5cm	63 (60.0%)	23 (56.1%)	40 (62.5%)	0.545	64 (62.1%)	9 (60.0%)	55 (62.5%)	0.854
	>5cm	42 (40.0%)	18 (43.9%)	24 (37.5%)		39 (37.9%)	6 (40.0%)	33 (37.5%)	
Tumor number	single	72 (68.6%)	32 (78.0%)	40 (62.5%)	0.131	71 (68.9%)	13 (86.7%)	58 (65.9%)	0.138
	multiple	33 (31.4%)	9 (22.0%)	24 (37.5%)		32 (31.1%)	2 (13.3%)	30 (34.1%)	
Vascular invasion	No	76 (72.4%)	33 (80.5%)	43 (67.2%)	0.206	74 (71.8%)	11 (73.3%)	63 (71.6%)	0.347
	Microvascular	19 (18.1%)	4 (9.8%)	15 (23.4%)		19 (18.4%)	4 (26.7%)	15 (17.0%)	
	Macrovascular	10 (9.5%)	4 (9.8%)	6 (9.4%)		10 (9.7%)	0	10 (11.4%)	
T-stage	1	60 (57.1%)	28 (68.3%)	32 (50.0%)	0.275	59 (57.3%)	10 (66.7%)	49 (55.7%)	0.491
	2	24 (22.9%)	6 (14.6%)	18 (28.1%)		24 (23.3%)	2 (13.3%)	22 (25.0%)	
	3	18 (17.1%)	6 (14.6%)	12 (18.8%)		17 (16.5%)	2 (13.3%)	15 (17.0%)	
	4	3 (2.9%)	1 (2.4%)	2 (3.1%)		3 (2.9%)	1 (6.7%)	2 (2.3%)	
Edmondson	I	4 (3.8%)	2 (4.9%)	2 (3.1%)	0.656	5 (4.9%)	0	5 (5.7%)	0.391
	II	55 (52.4%)	21 (51.2%)	34 (53.1%)		55 (53.4%)	9 (60.0%)	46 (52.3%)	
	III	40 (38.1%)	17 (42.5%)	23 (35.9%)		37 (35.9%)	4 (26.7%)	33 (37.5%)	
	IV	6 (5.7%)	1 (2.4%)	5 (7.8%)		6 (5.8%)	2 (13.3%)	4 (4.5%)	
Recurrence	Yes	34 (34.0%)	12 (29.3%)	23 (35.9%)	0.479	35 (34.0%)	4 (26.7%)	31 (35.2%)	0.518
	No	70 (66.7%)	29 (70.7%)	41 (64.1%)		68 (66.0%)	11 (73.3%)	57 (64.8%)	
Death	Yes	11 (10.5%)	5 (12.2%)	6 (9.4%)	0.747	11 (10.7%)	3 (20.0%)	8 (9.1%)	0.199
	No	94 (89.5%)	36 (87.8%)	58 (90.6%)		92 (89.3%)	12 (80.0%)	80 (90.9%)	

### Clinicopathological and molecular details of 8 patients with recurrent HCC

**Figure 1:**
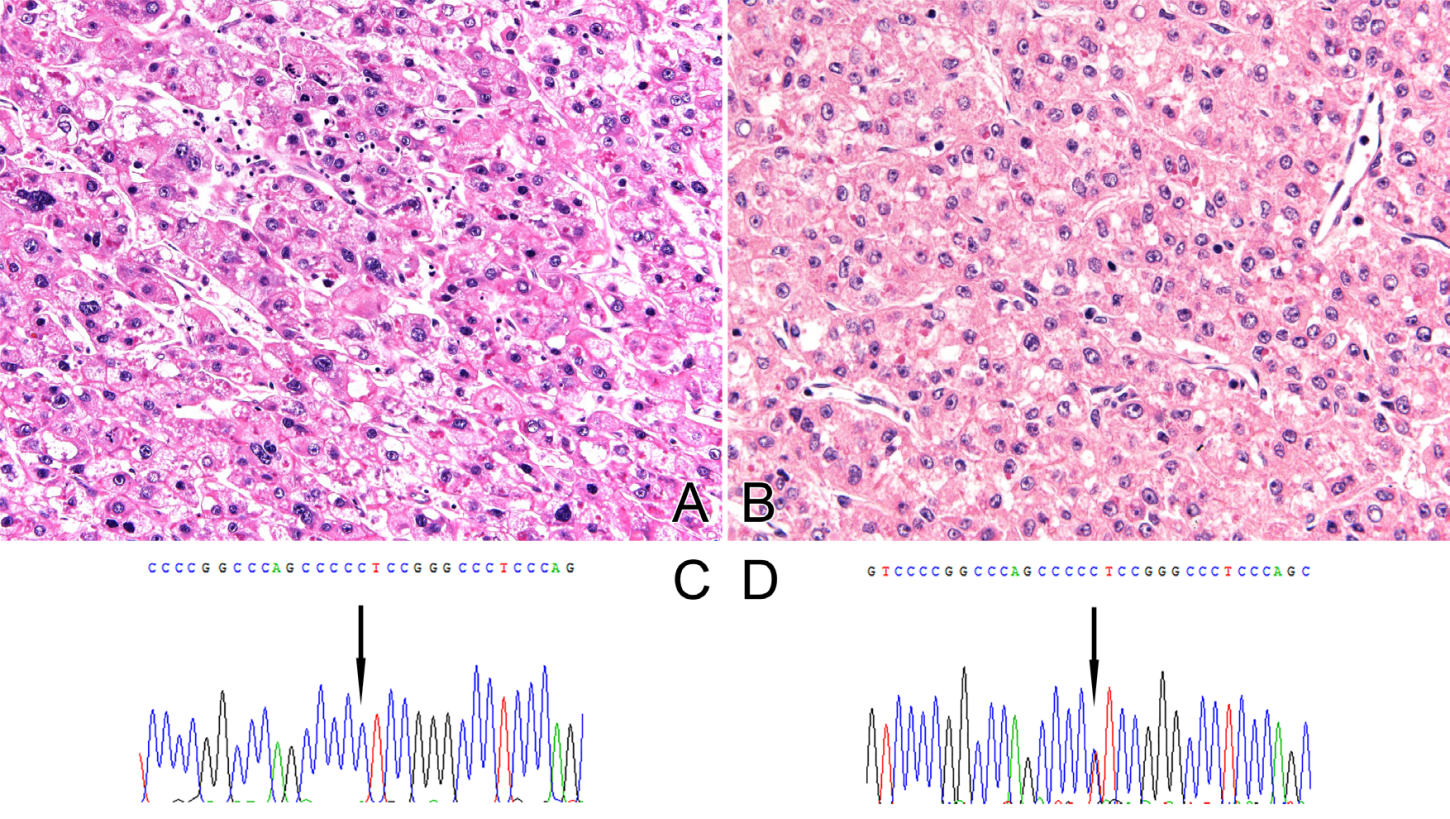
Examples of discordance in *TERT* mutation status between primary and corresponding recurrent HCC **A.** Histology of primary HCC with ES grade 3 and **B.** electropherogram of *TERT* C228T mutation **C.** Histology of recurrent HCC with ES grade 2 and **D.** electropherogram of *TERT* C228T mutation

We also analyzed 8 patients for which primary and corresponding recurrent HCC tumor tissues were available. Clinical details of the 8 patients are presented in Table 3. All but 1 case had chronic liver disease related to HBV infection. Two patients died due to HCC. Pathological and molecular details of the 8 patients are presented in Table 4. The clinicopathological and mutational status associated with primary and recurrent HCCs were compared. In 2 cases, discordance in *TERT* promoter mutation status was observed among primary and corresponding recurrent HCC. The mutation status of the 2 patients was changed from wild to mutant type at recurrence (Figure 1). All patients with discordant cases relapsed within 2 years, which is considered early relapse. Between 2 patients, 1 patient had multiple tumors and microvascular invasion at first presentation. On the other hand, all patients maintained the same *CTNNB1* mutational status.

**Table 3:** Clinical details in 8 patients with recurrent HCC

Case No.	Sex	Age at diagnosis	Site (primary/recurrent)	Cause	Underlying cirrhosis	Time to recurrent (month)	F/U duration after relapse	Outcome
1	M	52	Liver/Liver	HBV	Yes	17	40	Death
2	M	56	Liver/Liver	HBV	Yes	13	44	Alive
3	M	50	Liver/Liver	HBV	No	17	74	Death
4	M	53	Liver/Liver	HBV	No	2	5	Alive
5	M	74	Liver/Liver	HBV	No	13	19	Alive
6	F	56	Liver/Liver	HBV	Yes	6	28	Alive
7	M	79	Liver/Liver	HCV	Yes	10	4	Alive
8	M	27	Liver/Liver	HBV	No	7	29	Alive

**Table 4:** Pathological and molecular detains in 8 patients with recurrent HCC

Case No.	Stage (primary)	ES grade (primary/recurrent)	Vascular invasion (primary/recurrent)	Tumor multiplicity (primary/recurrent)	*TERT* mutation (primary/recurrent)	*CTNNB1* mutation (primary/recurrent)
1	4	3/4	Micorvascular/Micorvascular invasion	No/No	Wild/Wild	Mutant/Mutant
2	2	3/2	No / No invasion	Yes/No	Wild/ND	Wild/Wild
3	1	3/2	No / No invasion	Yes/Yes	Wild/Wild	Wild/Wild
4	3a	2/3	Macrovascular / No invasion	Yes/Yes	Wild/Wild	Wild/ND
5	2	3/2	Microvascular / No invasion	Yes/No	Wild/Mutant	Wild/Wild
6	2	2/2	No / No invasion	No/No	Wild/Mutant	Wild/Wild
7	2	3/3	No / No invasion	Yes/Yes	Mutant/Mutant	Wild/Wild
8	1	3/3	No / No invasion	No/No	Wild/Wild	Wild/Wild

### *TERT* promoter mutation and *CTNNB1* mutation in HCC and preneoplastic lesions

**Figure 2:**
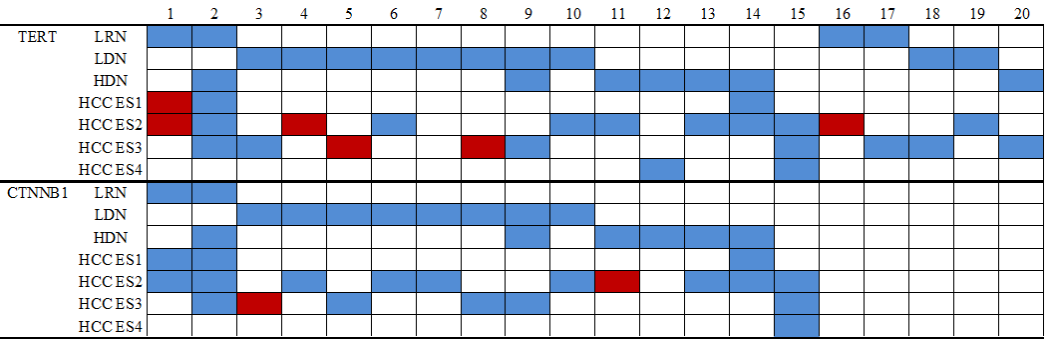
Heat map of *TERT* promoter and *CTNNB1* mutations Each case is represented by a single column.

Moreover, we screened 15 patients with primary HCC and matched LRN, LDN, and HDN tissues available. The heat map of the *TERT* promoter mutations and *CTNNB1* mutations are presented in Figure 2. All but 1 case had liver cirrhosis related to HBV infection. The mutation status of preneoplastic cirrhotic lesion to HCC was evaluated. *TERT* promoter mutations were detected in HCCs, and not observed in the matched LRN and LDN. Similarly, *CTNNB1* mutations were detected in HCCs, and not observed in the matched LDN and HDNs.

In cases with multiple HCCs, *TERT* promoter and *CTNNB1* mutation status did not differ based on the ES grade. The mutation status also did not differ in single HCC showing different ES.

## DISCUSSION

We evaluated the frequency of *TERT* promoter and *CTNNB1* mutations in 146 hepatocellular nodules including HCAs, LRNs, LDNs, HDNs, HCCs, and combined HCCs and cholangiocarcinomas. Among the 121 HCC cases (including recurrent tumors in a patient and multiple tumors showing a different ES grade in a patient), 37.8% (45 of 119) and 13.8% (16 of 116) harbored *TERT* promoter mutations and *CTNNB1* mutations, respectively. Of 109 patients with HCC, 41 (39.0%) and 15 (14.6%) harbored *TERT* and *CTNNB1* mutations, respectively. In HCC, previously published data show that the frequency of *TERT* promoter and *CTNNB1* mutations were 44~59% and 20~40%, respectively. The frequencies of these mutations in the present study were slightly lower compared to those reported in previous studies. These may have been due to the higher prevalence of HBV-related HCC in Korea. In the present study, the frequency of HCCs related to HBV infection was 74.3% (78/109), which is much higher compared to those reported by previous studies upon analyses of these mutations. Nault et al. [8] identified *TERT* promoter mutations in HCC to be more common in non-related HBV. It is also known that the insertion of HBV DNA into the *TERT* promoter induces telomerase transcription [20, 21]. Several studies reported that *CTNNB1* mutations were identified at higher frequencies in HCCs related to HCV infection [22–24]. In our study, *TERT* promoter mutations were significantly more common in HCC related to HCV infection (5/6; 83.3%) compared to tumors of other etiologies; however, *CTNNB1* mutations did not differ between HBV-related HCC and other etiologies-related HCC.

We did not identify a significant association between *TERT* promoter mutations and *CTNNB1* mutations. However, the limited number of HCV-related HCC cases analyzed, restrains the conclusion that *TERT* promoter mutations might be significantly associated with WNT pathway gene alterations, such as *CTNNB1* in HCV-related HCC and non-viral HCCs [6, 8].

The absence of *TERT* promoter mutations in our series of 15 HCAs without malignant transformation is consistent with a previous study, which reported that *TERT* promoter mutations are common in HCAs with malignant transformation [8].

In this study, *TERT* promoter and *CTNNB1* mutations were not identified in LRN, but were identified in matched HCC, indicating that they were somatic mutations.

Recently, *TERT* promoter mutations were identified in 6% of LDNs, 19% of HDNs, and 61% of early HCCs [25]. The prevalence of mutations gradually increased with the degree of dysplasia, indicating *TERT* promoter mutations were the earliest genetic events in the multistep process of hepatocarcinogenesis [8, 25]. However, we never identified any mutations in the precursor lesions of HCC. One reason for the observed discrepancy could be attributed to the small number of dysplastic nodules studied. A further reason this discrepancy could be diagnostic confusion concerning equivocal nodular lesions in the cirrhotic liver. Regarding the pathological and clinical diagnosis of equivocal nodular lesions found in cirrhotic liver, there is a discrepancy in interpretation [26]. Although the majority of nodular lesions are HCC in cirrhotic background, equivocal nodules in which it is difficult to distinguish between well-differentiated HCC and high-grade dysplastic nodules. Therefore, a large study with precancerous lesions accurately diagnosed is warranted to resolve an issue. Another reason could be the differences in the ethnic population; a cohort enriched for HBV-related HCC patients.

Meanwhile, Park [27] et al. described that *CTNNB1* mutations were not found in precursor lesions of HCC and were not uniformly present in all tumor lesions, indicating that these mutations are late events in hepatocarcinogenesis.

Notably, we first described 2 cases of discordant *TERT* promoter mutations between primary and corresponding recurrent HCCs. Two patients with discordant cases had early relapses. In HCC, early recurrence might be largely related to metastasis from the primary tumor, while late recurrence might be due to *de novo* tumors from non-tumoral lesions on a cirrhotic background that are independent of resected primary tumors. From our findings, we could speculate two hypotheses. *TERT* promoter mutations in recurrent HCCs could be from minor subclones, which were not detected at the initial presentation by Sanger sequencing technology. Conventional sequencing might have missed intratumoral heterogeneity by representing only the dominant clone. *TERT* promoter mutations are not acquired in proportion to cancer progression; however, are already encoded in the primary tumors. Even though we identified that there was no heterogeneous distribution of *TERT* promoter and *CTNNB1* mutations from multiple regions in a single HCC, intratumoral genetic heterogeneity could not be fully ruled out. It is known that HCC shows morphological and immunophenotypical heterogeneity, indicating that HCC can display intratumoral genetic heterogeneity. Intratumoral genetic heterogeneity may indicate tumor evolution, adaptation to environmental stress, and response to treatment. Furthermore, intratumoral genetic heterogeneity is a practical challenge with clinical implications in the era of targeted therapy. Therefore, understanding of intratumoral genetic heterogeneity is crucial in clinical management. Recently, Friemel [28] et al. identified that heterogeneous intratumoral mutational status of *TP53* and *CTNNB1* mutations was present in 22% of HCCs. However, there are no data regarding intratumoral heterogeneity of other somatic mutations.

It could also be assumed that the development of a tumor cell clone showing *TERT* promoter mutations in recurrence was not present in the primary HCC. It is thought that *TERT* promoter mutations were acquired during their progression, not as early events in HCC. However, this is in contrast to the current view that *TERT* promoter mutations are among the earliest genetic alterations involved in malignant transformation [25].

We are fully aware that the number of cases in our study is too small to reach a definitive conclusion. Furthermore, these speculations could be supported by deep sequencing to detect mutations with low frequencies and by multiregional sequencing, which reveals cancer genome obtained from multiple regions in a single tumor. The role of *TERT* promoter mutations in hepatocarcinogenesis, and the pathogenesis of tumor progression remain to be functionally investigated.

In conclusion, we identified *TERT* promoter mutations to be the most frequent somatic genetic alterations in HCC, indicating that they play a pivotal role in hepatocarcinogenesis. Furthermore, we suggest the possibility of intratumoral genetic heterogeneity of *TERT* promoter mutations in HCC through the discordance in *TERT* promoter mutations between primary and corresponding recurrent HCCs. Further studies in a large cohort are needed to support our observations.

## MATERIALS AND METHODS

### Patient selection and characteristics

Study approval was obtained from the Institutional Review Board of Konkuk University Medical Center (KUH 1210050). This study included 111 patients who underwent hepatectomy for HCC (*n* = 109)/HCA (*n* = 2), and 7 patients who underwent liver needle biopsy for HCA between 2006 and 2016 at the Konkuk University Medical Center in Seoul, Korea. A total of 156 liver nodules from 113 patients were evaluated, including 4 large regenerating nodules (LRNs), 10 low grade dysplastic nodules (LDNs), 7 high-grade dysplastic nodules (HDNs), 9 HCAs, 123 HCCs, and 3 combined HCAs and cholangiocarcinomas. Tumor stage was determined according to the American Joint Committee on Cancer (AJCC) staging system. The histologic grade of differentiation was determined on the basis of Edmondson and Steiner (ES) classification [29]. Histologic grades of worst differentiation were recorded. All of the 156 hematoxylin and eosin-stained slides were reviewed; representative tumor tissue samples were selected from each case.

### DNA extraction

DNA was extracted from formaldehyde fixed-paraffin embedded (FFPE) tumor tissue. Briefly, 100 μL of the MultiTech DNA extraction solution containing 16 mM (NH_4_)_2_SO_4_, 50 mM Tris-HCl, pH 8.5, 1 mM EDTA pH 8.0, and 0.5% Tween-20 was added to dissected cells in a 1.5-mL microcentrifuge tube. Next, proteinase K (Takara Bio. Inc., Shiga, Japan) was added to a final concentration of 200 μg/mL, and the tissue was digested at 56°C for 1 h. Following digestion, Chelex-100 (Bio-Rad, CA, USA) was added to a final concentration of 10% in the PCR tubes, and the tubes were heated to 100°C for 10 min in a dry bath incubator (Major Science, New Taipei City, Taiwan). After gentle shaking, the tubes were centrifuged at 12,000 ×g for 10 min for DNA elution [30].

### Identification of TERT promoter mutations by PCR amplification and direct sequencing

Standard PCR was carried out for genetic sequencing to identify *TERT* promoter mutations. Briefly, a fragment of the *TERT* promoter was amplified by PCR on genomic DNA using primers 5′-AGTGGATTCGCGGGCACAGA-3′ (sense) and 5′-CAGCGCTGCCTGAAACTC-3′ (antisense). About 40-50 ng of genomic DNA was used in the PCR, which was carried out with an initial denaturation step at 95°C for 3 min, followed by ten cycles of 95°C denaturation for 30 sec, 58~62°C annealing for 30 sec, and 68°C elongation for 1 min. This was followed by 30 cycles under the same settings, except for the elongation step that was modified to continue for an additional 5 sec in each cycle. The PCR was completed with a final elongation step at 68°C for 7 min. The PCR products were subsequently subjected to a sequencing reaction using the BigDye Terminator Cycle Sequencing Ready Reaction Kit (Applied Biosystems, Foster City CA, USA). Sequencing was performed using the following PCR reaction settings: 35 cycles of denaturation for 60 sec at 96°C, annealing for 5 sec at 50°C, and elongation for 4 min at 60°C. PCR was performed in a final volume of 10 μL, containing 2.5 μL of 70 ng/μL PCR product, 0.5 μL of sequencing primer (3 picomoles), 5X buffer, and AmpliTaq DNA polymerase. DNA sequence was then read on an ABI 3730XL DNA Analyzer (Applied Biosystems). Sequences were aligned using the ContigExpress alignment program (InforMax, Frederick MD, USA).

### Identification of CTNNB1 mutations by PCR amplification and direct sequencing

In order to amplify exon 3 of the *CTNNB1* gene, 2 PCR methods were used with catenin F (CCT GGC TAT CAT TCT GCT TTT C), catenin R (TCA AAA CTG CAT TCT GAC TTT CA), Beta GF (CCA ATC TAC TAA TGC TAA TAC TG), and Beta GR (CTG CAT TCT GAC TTT CAG TAA GG) primers. The first PCR method using catenin F and catenin R primers was programmed as follows: initial denaturation at 94°C for 2 min, 35 cycles of denaturation at 94°C for 30 sec, annealing at 58°C for 30 sec, and elongation at 72°C for 1 min, followed by a final extension cycle at 72°C for 10 min. The second method used was Touchdown PCR (TD-PCR) with Beta GF and Beta GR primers. The PCR was programmed as follows: initial step of denaturation at 95°C for 2 min, 20 cycles of denaturation at 95°C for 30 sec, annealing temperature starting at 69°C for 30 sec (decreasing by 1°C /2 cycles), and elongation at 72°C for 1 minute. This was followed by 30 cycles of denaturation at 95°C for 30 sec, annealing at 59°C for 30 sec, elongation at 72°C for 1 min, and a final elongation cycle at 72°C for 10 min. Finally, the PCR products were purified and sequenced (Macrogen, Korea). Sequencing reactions were performed in the DNA Engine Tetrad 2 Peltier Thermal Cycler (BIO-RAD) using the ABI BigDye (R) Terminator v3.1 Cycle Sequencing Kit (Applied Biosystems), following the protocols supplied by the manufacturer. Single pass sequencing was performed on each template using F primer. The fluorescently labeled fragments were purified using the method recommended by Applied Biosystems, as it removed the unincorporated terminators and dNTPs. The samples were subjected to electrophoresis in an ABI 3730xl DNA Analyzer (Applied Biosystems).

### Statistical analysis

For the analysis of the relationship between clinicopathological factors and presence of *TERT* C228T*/CTNNB1* mutations, Pearson chi-squared test and Fisher's exact test were used. Linear-by-linear testing was used to examine the associations between T stage, AJCC stage, and *TERT* C228T*/CTNNB1* mutations. The time to recurrence-free survival was defined from the day of first surgery until recurrence. All tests were 2-sided, with *P* < 0.05 considered as statistically significant. All statistical analyses were performed using the SPSS software (SPSS Inc., Chicago IL, USA).
